# Demethylation of miR‐195 suppresses prostate cancer cell proliferation, migration and invasion

**DOI:** 10.1002/2211-5463.12799

**Published:** 2020-03-09

**Authors:** Xiaokun Ma, Liyuan Zou, Zhanhong Chen, Xing Li, Li Wei, Xiangyuan Wu

**Affiliations:** ^1^ Department of Medical Oncology The Third Affiliated Hospital of Sun Yat‐sen University Guangzhou China; ^2^ Department of Prevention and Health Care The Third Affiliated Hospital of Sun Yat‐sen University Guangzhou China

**Keywords:** methylation, miR‐195, prostate cancer, tumor suppressor

## Abstract

Prostate cancer (PCa) is the most prevalent cancer among men and the second leading cause of tumor‐associated deaths worldwide, with increasing incidence rates over the last 10 years. Recently, miR‐195 was reported to be hypermethylated at its promoter CpG island and down‐regulated in hepatocellular carcinoma. However, the function of miR‐195 and the underlying mechanisms in PCa remain unknown. Here, we report that a significant down‐regulation of microRNA‐195 (miR‐195) in PCa tissues and cell lines was associated with promoter methylation status. Overexpression of miR‐195 significantly suppressed cell proliferation, migration, invasion and epithelial–mesenchymal transition (increased E‐cadherin and decreased N‐cadherin) in PCa cells. We further demonstrated that transfection with a miR‐195 inhibitor reversed the inhibitory effect of the DNA methyltransferase inhibitor 5‐azacytidine on the proliferation, migration and invasion ability of PCa cells. In summary, our findings suggest that miR‐195 may function as a crucial tumor suppressor in PCa.

## Graphical abstract

The tumor environment of prostate cancer promotes the methylation of microRNA‐195 (miR‐195) promoter, thereby inhibiting the expression of miR‐195, and the methyltransferase inhibitor 5‐azacytidine can reverse the methylation, promote the expression of miR‐195, and inhibit the proliferation, migration and invasion of tumor cells.

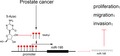

**Abbreviations**
5‐AzaC: 5‐azacytidine
BSP: bisulfite sequencing PCR
CCK‐8: Cell Counting Kit‐8
E‐cadherin: epithelial marker
EMT: epithelial–mesenchymal transition
HCC: hepatocellular carcinoma
MSP: methylation‐specific PCR
miR‐195: microRNA‐195
miRNA: microRNA
NC: negative control
N‐cadherin: mesenchymal marker
PCa: prostate cancer
PI: propidium iodide

Prostate cancer (PCa) is the most prevalent cancer among men and the second leading cause of tumor‐associated deaths worldwide, with increasing incidence rates over the last 10 years 1, 2. Early PCa may be managed by radical prostatectomy, but a majority of patients eventually experience metastatic disease 3. Prostate tumors contain marked heterogeneous phenotypes with various survival rates from 2–3 years to 10–20 years, reflecting the potential genomic diversity 4. Nowadays, involvement of numerous genes in the regulation of PCa has attracted great public interest, but the molecular mechanisms underlying this malignancy have not been fully elucidated.

Widespread epigenomic dysregulation events as a hallmark of tumorigenesis frequently occur in PCa according to recent studies 5, 6, 7. As one of the epigenetic alterations, aberrant tumor‐specific DNA methylation causes gene expression repression alterations, which are an emerging class of therapeutic targets for prostate carcinogenesis 8, 9, 10, 11. As noncoding small RNA molecules (19–25 nucleotides), microRNAs (miRNAs) play a vital role in cellular functions, including proliferation, apoptosis and invasion in human diseases, through interacting with mRNAs 12, 13. Up‐regulated miRNAs function as oncogenes targeting tumor suppressor genes 14, 15, whereas down‐regulated miRNAs exert as tumor suppressors targeting oncogenes in PCa 16, 17, 18. Notably, it has been suggested that inhibition of tumor suppressor miRNAs by DNA methylation is a widely accepted mechanism in the development of tumors, including PCa 19.

MicroRNA‐195 (miR‐195) is mapped to chromosome 17p13.1 and is one of the miR‐15/107 family members with a similar sequence (AGCAGC) 20. Accumulating evidence suggests that abnormal expression of miR‐195 may contribute to the pathogenesis of many diseases, including Alzheimer’s disease 21, heart failure 22 and cerebral ischemia 23. Recently, miR‐195 was identified as being down‐regulated in several tumor tissues, such as breast cancer 24, cervical cancer 25 and hepatocellular carcinoma (HCC) 26, which acts as tumor suppressor via targeting the mRNAs of multiple proteins. In particular, miR‐195 is frequently reported to suppress cell proliferation and metastasis by targeting RPS6KB1 27, BCOX1 28, fibroblast growth factor 2 (FGF2) 29 and PRR11 30, as well as enhance drug resistance in PCa 31, 32. Interestingly, He et al. 33 found that miR‐195 was silenced by promoter CpG island hypermethylation in HCC 33.

In this study, we first analyzed the profile of miR‐195, as well as the DNA methylation of its promoter in PCa tissues. Then, we explored the impact of miR‐195 on cell proliferation, migration, invasion and epithelial–mesenchymal transition (EMT), and evaluated whether these effects were correlated with DNA methylation of the miR‐195 promoter.

## Materials and methods

### Clinical specimens

A total of 30 paired surgical resected PCa tissues and adjacent noncancerous tissues were obtained from the Third Affiliated Hospital of Sun Yat‐sen University (Guangdong, China) between October 2017 and September 2018. All of the patients were confirmed not to receive any preoperative chemotherapy or radiotherapy. All collected specimens were snap frozen in liquid nitrogen promptly and kept at −80 °C for further experiments. Written informed consent was signed by all patients before enrollment in clinical trials. The current study was approved by the Ethics Committee of the Third Affiliated Hospital of Sun Yat‐sen University (Guangdong, China), and the study conformed to the standards set by the Declaration of Helsinki.

### Methylation‐specific PCR

Genomic DNA was isolated from tissues or cell lines by DNA Tissue Kit (Qiagen, Duesseldorf, Germany) and applied to bisulfite modification by using the EZ DNA Methylation‐Gold Kit (Zymo Research, Irvine, CA, USA) according to the instruction’s observation protocol. For methylation‐specific PCR (MSP), bisulfite‐converted genomic DNA was used as a template, and specific primers design was carried out by using methyl primer express v1.0. software (Applied Biosystems Inc., Foster City, CA, USA).

### Cell culture and transfection

Four PCa cell lines, LNCAP, PC‐3, DU145 and 22RV1, and a normal prostate epithelial cell line, RWPE‐1, were purchased from American Type Culture Collection (ATCC, Manassas, VA, USA). LNCAP, PC‐3 and 22RV1 cells were cultured in RPMI‐1640 medium (Gibco, Carlsbad, CA, USA). DU145 cells were cultured in the Dulbecco’s modified Eagle’s medium (Life Technologies, Carlsbad, CA, USA). RWPE‐1 cells were grown in defined keratinocyte serum‐free medium (Invitrogen, Carlsbad, CA, USA). All cells were grown in medium containing 10% FBS (Gibco) and incubated at 37 °C under a humidified atmosphere of 5% CO_2_.

For cell transfection, two cell lines (PC‐3 or DU145) were cultured in six‐well plates overnight until reaching 70–80% confluence. RiboBio (Guangzhou, China) has synthesized inhibitor of miR‐195 inhibitor, mimics of miR‐195 (miR‐195 inhibitor: 5′‐GCCAAUAUUUCUGUGCUGCUA‐3′; miR‐195 mimics: 5′‐UAGCAGCACAGAAAUAUUGGC‐3′, 5′‐CAAUAUUUCUGUGCUGCUAUU‐3′) and corresponding negative control (miR‐NC, 5′‐CAGUACUUUUGUGUAGUACAA‐5′). At a final concentration of 50 nm, transfection was carried out using the Lipofectamine 2000 reagent (Invitrogen) according to the manufacturer’s protocols.

### 5‐Azacytidine treatment

A density of 4 × 10^5^ cells/well of PC‐3 or DU145 cells were seeded in six‐well culture dishes and incubated with 2.5 μm 5‐azacytidine (5‐AzaC; Sigma‐Aldrich, St. Louis, MO, USA). After 72‐h treatment, cells were collected for bisulfite sequencing PCR (BSP) and further *in vitro* experiments.

### BSP

For BSP, bisulfite‐converted genomic DNA was amplified with primers specific for BSP (designed using methyl primer express v1.0, Applied Biosystems Inc) and then cloned into pMD19‐T (TaKaRa, Dalian, China). Subsequently, five independent clones from each sample were sequenced on iCycler Thermal Cycler (Eppendorf, Hamburg, Germany).

### Quantitative real‐time PCR

Total RNA was extracted from tissues or cell lines using TRIzol reagent (Invitrogen), and cDNA was generated using random primers. Using the LightCycler 480 II Real‐Time PCR system (Roche Diagnostics, Basel, Switzerland), the level of miR‐195 was evaluated with SYBR Green PCR Master Mix of Hairpin‐miRNA RT‐PCR Quantitation Kit (GenePharma, Shanghai, China). Relative quantification of miR‐195 was analyzed using the $2^{-\Delta \Delta C_{t}}$ method with U6 snRNA as endogenous control. The primer sequences used were as follows: miR‐195 forward: 5′‐ACACTCCAGCTGGGTAGCAGCACAGAAATATT‐3′, reverse: 5′‐CTCAACTGGTGTCGTGGAGTCGGCAATTCAGTTGAGGCCAATA‐3′; U6 forward: 5′‐CTCGCTTCGGCAGCACA‐3′, reverse: 5′‐AACGCTTCACGAATTTGCGT‐3′; epithelial marker (E‐cadherin) forward: 5′‐CGAGAGCTACACGTTCACGG‐3′, reverse: 5′‐GGGTGTCGAGGGAAAAATAGG‐3′; mesenchymal marker (N‐cadherin) forward: 5′‐TCAGGCGTCTGTAGAGGCTT‐3′; reverse: 5′‐ATGCACATCCTTCGATAAGACTG‐3′; glyceraldehyde‐3 phosphate dehydrogenase forward: 5′‐GGAGCGAGATCCCTCCAAAAT‐3′, reverse: 5′‐A GGCTGTTGTCATACTTCTCATGG‐3′.

### Cell proliferation assay

PC‐3 or DU145 cells from different groups were grown in 96‐well plates (2 × 10^3^ cells/well) and cultured overnight. At multiple time points (24, 48, and 72 h, respectively), 10 μL of Cell Counting Kit‐8 solution (CCK‐8; Dojindo, Kumamoto, Japan) was added into each well, and the cells were cultured for another 2 h at 37 °C. The absorbance (*A*) at 450 nm (*A*_450 nm_) was determined using a microplate reader (Bio‐Tek ELX800; Winooski, VT, USA).

### Cell apoptosis analysis

Flow cytometry assay was performed for cell apoptosis detection. In brief, approximately 3 × 10^5^ cells from different groups were harvested, washed two times in PBS and then orderly stained with FITC‐Annexin V and propidium iodide (PI) according to the FITC‐Annexin V Apoptosis Detection Kit (BD Biosciences, San Jose, CA, USA). Stained cells were analyzed by fluorescence‐activated cell sorter using FACScan (BD Biosciences) equipped with cell quest 3.0 software (BD, Franklin Lakes, NJ, USA).

### Transwell assay

Transwell assay was carried out in PC‐3 or DU145 cells from different groups using a 24‐well Transwell chamber with 8‐µm pore size (Costar; Corning, Inc., Corning, NY, USA) without coated Matrigel (BD Biosciences) for cell migration or with coated Matrigel for cell invasion. In brief, 3 × 10^5^ cells were transferred to the top chamber, and the chemoattractant (the medium containing 10% FBS) was added to the lower chamber. Following 24‐h incubation, 4% paraformaldehyde was used for fixation of those cells that migrated into the lower chamber; then, the cells were stained by 0.1% crystal violet; and finally, the cell counting was performed on a microscope (Olympus Corporation, Tokyo, Japan).

### Western blot analysis

Total cellular protein was extracted from cells using ice‐cold radioimmune precipitation assay buffer (Beyotime, Shanghai, China), and the concentration of protein was evaluated by the BCA protein assay kit (Beyotime). Twenty micrograms of protein was separated by 10% SDS/PAGE and then transferred to polyvinylidene difluoride membranes (Merck Millipore, Darmstadt, Germany). After blocking with 5% nonfat milk, the membranes were incubated with primary antibodies against E‐cadherin, N‐cadherin and glyceraldehyde‐3 phosphate dehydrogenase overnight at 4 °C, followed by incubation with a secondary, horseradish peroxidase‐conjugated antibody (Cell Signaling Technology, Danvers, MA, USA) for 1 h at room temperature. Then, these protein bands were measured using an enhanced chemiluminescence detection kit (Pierce; Thermo Fisher Scientific, Inc, Basingstoke, United Kingdom). Glyceraldehyde‐3 phosphate dehydrogenase was used as an internal control.

### Statistical analysis

All experiments were carried out in at least triplicate. Analysis of statistical data was conducted with spss version 21.0 software (IBM Corp., Armonk, NY, USA). Data were expressed as mean ± SD. For comparison between two groups, Student’s *t*‐test was performed. For groups of more than three groups, one‐way ANOVA was performed. Statistical tests were considered significant when the *P*‐value was less than 0.05.

## Results

### miR‐195 expression was down‐regulated in PCa tissues and cell lines

**Figure 1:**
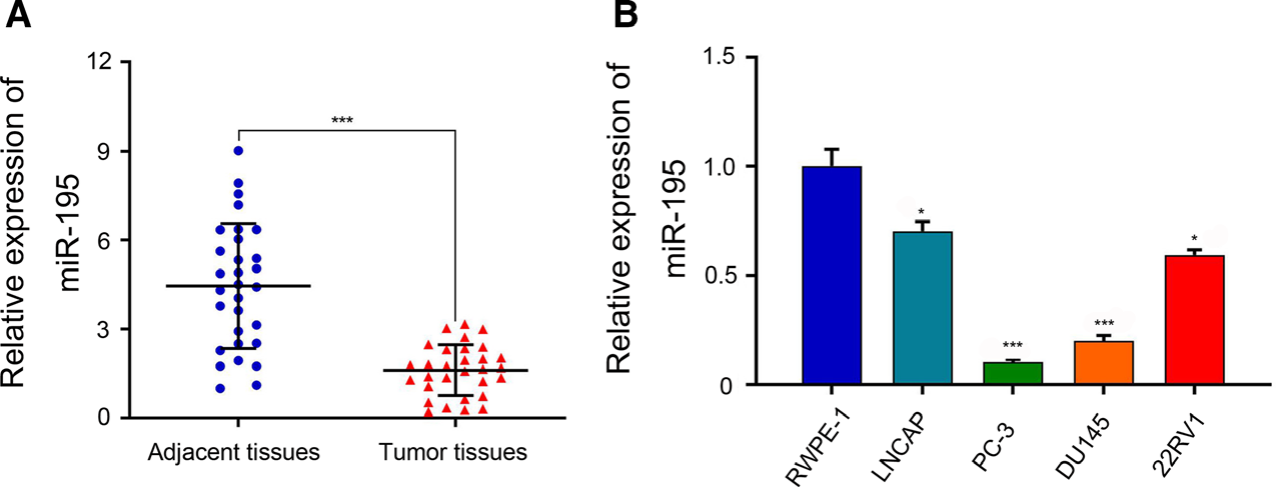
miR‐195 was down‐regulated in PCa tissues and cell lines. Quantitative real‐time PCR was performed to determine miR‐195 expression in (A) 30 paired tumor tissues and matched adjacent tissues, as well as in (B) PCa cell lines (LNCAP, PC‐3, DU145 and 22RV1) and one normal prostate epithelial cell line, RWPE‐1. The data are presented as the mean ± SD; *n* = 3; *P < 0.05, ***P < 0.001, compared with adjacent tissues or RWPE‐1 cells; two‐tailed Student’s *t*‐test.

To investigate the role of miR‐195 in PCa, we analyzed the relative expression of miR‐195 in 30 pairs of PCa and adjacent tissues using quantitative real‐time PCR. As shown in Fig. 1A, miR‐195 expression was dramatically down‐regulated in PCa tissues compared with paired adjacent tissues (*P* < 0.001). In a further analysis, endogenous expression of miR‐195 was determined in four PCa cell lines, LNCAP, PC‐3, DU145 and 22RV1, and a normal prostate epithelial cell line, RWPE‐1. All four PCa cell lines exhibited relatively low miR‐195 expression in comparison with RWPE‐1 cells (Fig. 1B).

### miR‐195 overexpression significantly suppressed malignant biological behaviors of PCa cells

**Figure 2:**
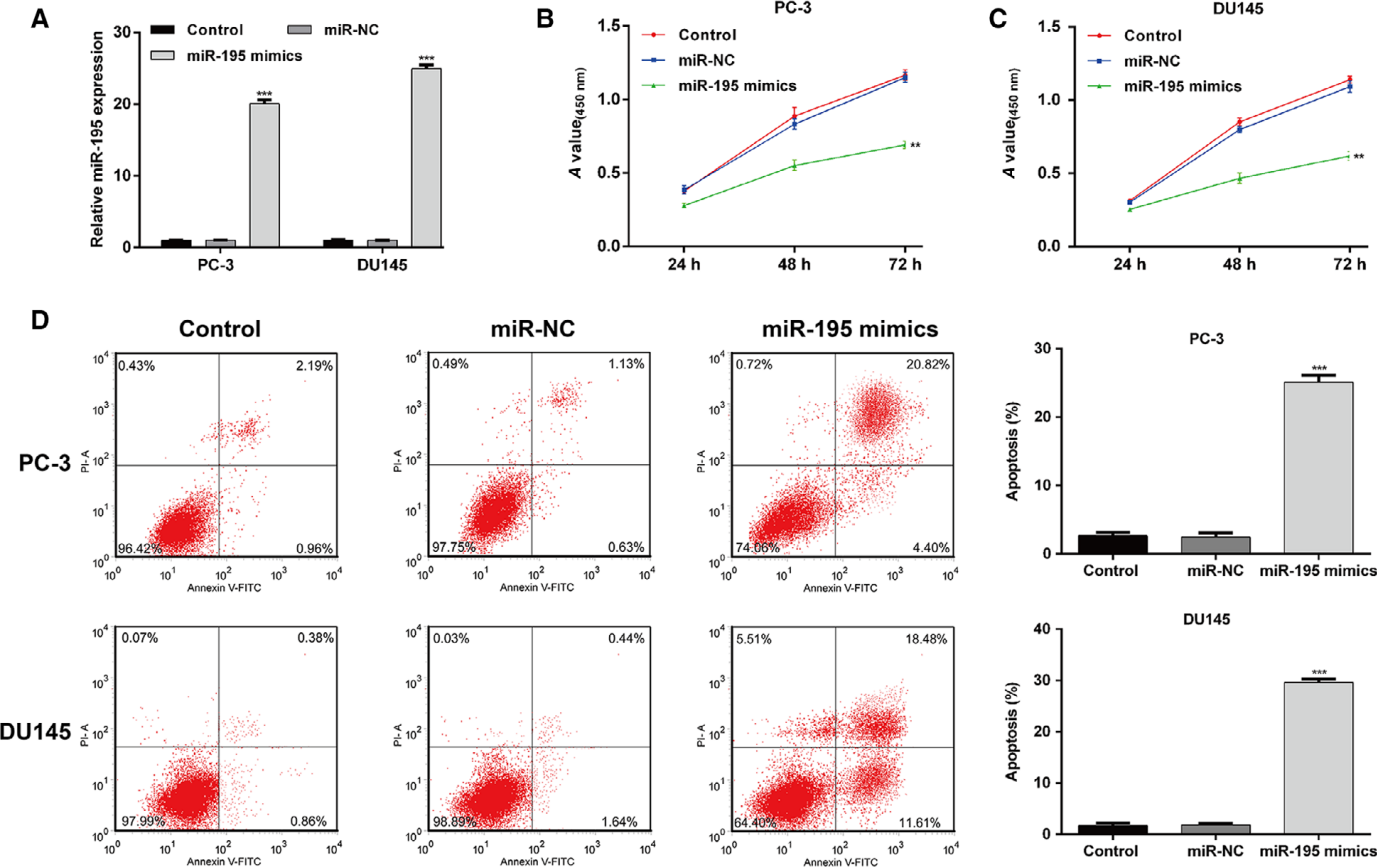
miR‐195 suppresses PCa cell proliferation in vitro. (A) miR‐195 expression was significantly up‐regulated after transfection with miR‐195 mimics in PC‐3 and DU145 cells. (B, C) CCK‐8 assay revealed that miR‐195 suppresses PCa cell proliferation. (D) Annexin V/PI double staining with flow cytometry analysis of PC‐3 and DU145 cells transfected with miR‐195 mimics. The data are presented as the mean ± SD; *n* = 3; **P < 0.01, ***P < 0.001, compared with miR‐NC; two‐tailed Student’s *t*‐test.

**Figure 3:**
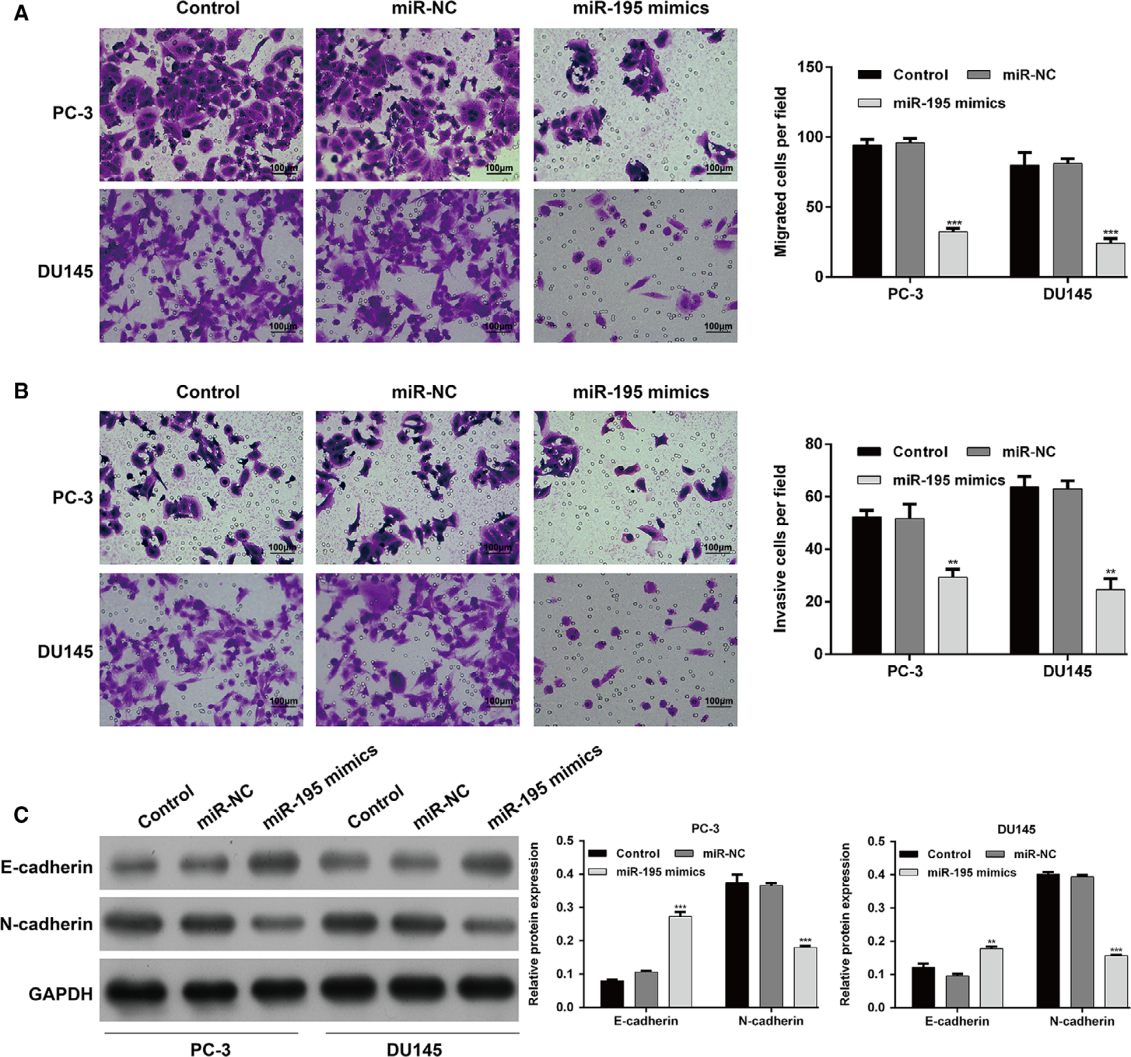
miR‐195 suppresses PCa cell migration and invasion in vitro. Transwell assay uncovered that miR‐195 repressed PCa (A) cell migration and (B) invasion ability. Original magnification ×100. (C) Western blot assay exhibited that up‐regulated miR‐195 increased the expression of E‐cadherin and inhibited the expression of N‐cadherin in PC‐3 and DU145 cells. Scale bars, 100 μm. The data are presented as the mean ± SD; *n* = 3; **P < 0.01, ***P < 0.001, compared with miR‐NC; two‐tailed Student’s *t*‐test.

We overexpressed miR‐195 in PCa cell lines by transfecting miR‐195 mimics into PC‐3 and DU145 cells with relatively lower miR‐195 expression. We demonstrated that miR‐195 expression profiles were significantly increased in both PC‐3 and DU145 cells after transfection with miR‐195 mimics (Fig. 2A, *P* < 0.001). Subsequently, a significant reduction in PC‐3 (Fig. 2B, *P* < 0.01) and DU145 (Fig. 2C, *P* < 0.01) cell proliferation was observed in the miR‐195 mimics group at 48 and 72 h. Annexin V/PI double staining revealed that the proportion of apoptosis was increased in PC‐3 and DU145 cells after transfection with miR‐195 mimics (Fig. 2D, *P* < 0.001). In addition, the protein level of Bax was significantly increased, whereas Bcl2 in PC‐3 and DU145 cells was decreased by miR‐195 overexpression (Fig. S1). As shown in Fig. 3A, miR‐195 mimics–transfected PC‐3 and DU145 cells underwent an obvious reduction in migratory activity (*P* < 0.001) and invasion ability (Fig. 3B, *P* < 0.01). Western blot analysis (Fig. 3C) exhibited that up‐regulation of miR‐195 increased the expression of E‐cadherin and inhibited the expression of N‐cadherin. These findings suggest that down‐regulated miR‐195 might be an important cause for uncontrolled PCa cell proliferation, migration and invasion.

### miR‐195 expression was regulated by DNA methylation of its promoter

**Figure 4:**
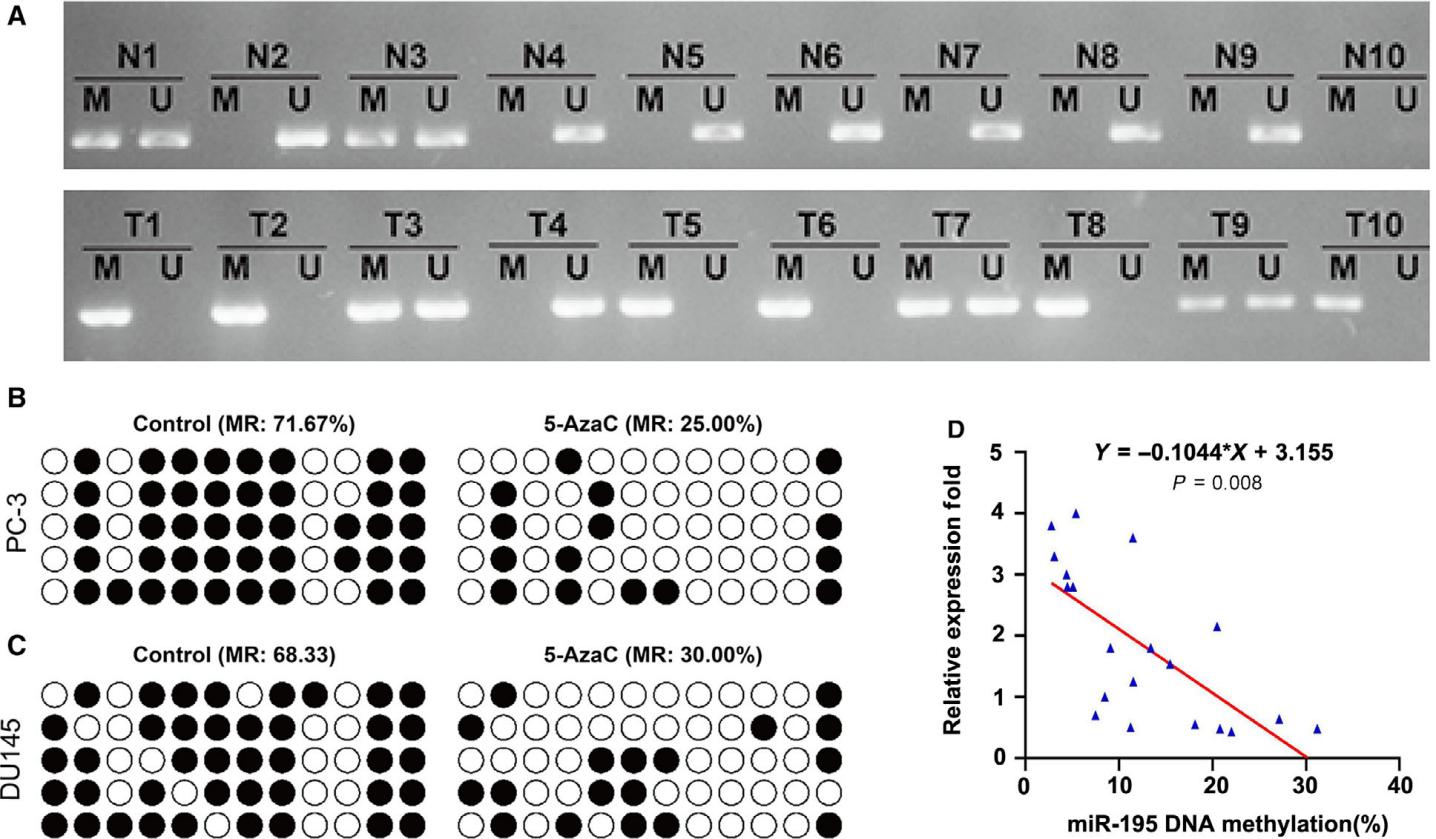
miR‐195 expression was regulated by DNA methylation of its promoter in PCa. (A) MSP analysis uncovered hypermethylation of the miR‐195 promoter in PCa tissues when compared with adjacent normal tissues. BSP analysis demonstrated that methylated CG sites were decreased in (B) PC‐3 and (C) DU145 cells after being treated with DNA methylation inhibitor 5‐AzaC. (D) The correlation between the methylation status and miR‐195 mRNA levels in the individual paired N and T samples. M, methylated; MR, methylation ratio; N, normal; T, tumor; Um, unmethylated.

To explore the upstream mechanism implicated in miR‐195 down‐regulation in PCa, we analyzed the DNA methylation status of miR‐195 promoter using MSP. As shown in Fig. 4A, hypermethylation of the miR‐195 promoter was frequently observed in tumor samples (8/10) in comparison with paired adjacent normal tissues (2/10). To validate the importance of promoter methylation in the regulation of miR‐195 levels, we treated PC‐3 and DU145 cells with DNA methylation inhibitor 5‐AzaC. BSP analysis demonstrated that 5‐AzaC treatment obviously decreased the methylated CG sites in both PC‐3 (Fig. 4B) and DU145 (Fig. 4C) cells. We analyzed the correlation between the methylation status and miR‐195 mRNA levels in the individual paired N and T samples, and found the methylation level was negatively related with the miR‐195 mRNA levels (Fig. 4D). Meanwhile, miR‐195 expression was detected by quantitative real‐time PCR. As shown in Fig. S2, the expression of miR‐195 was notably restored in these two PCa cell lines (*P* < 0.001).

### Elevated PCa cell progression was closely correlated with DNA methylation of the miR‐195 promoter

**Figure 5:**
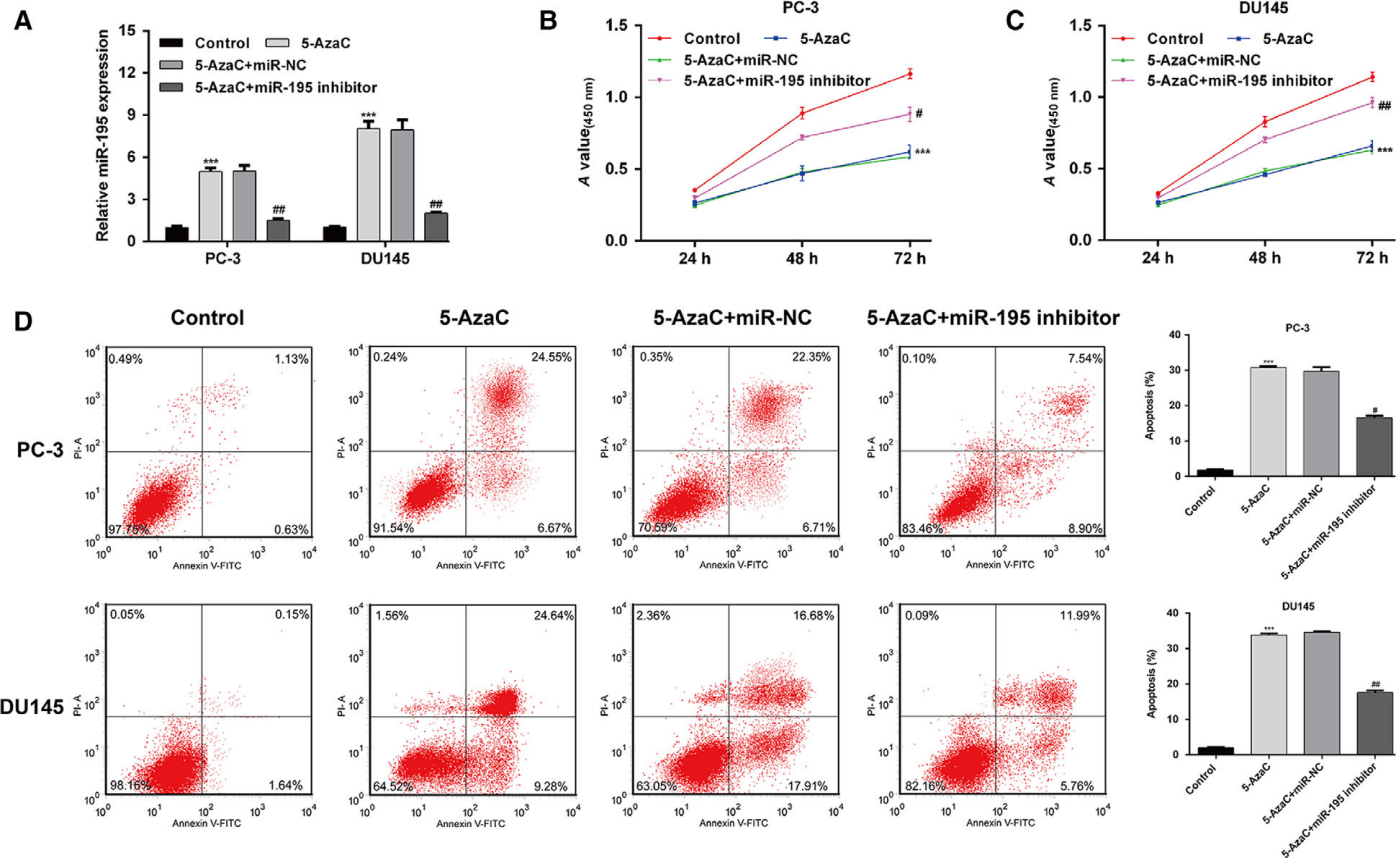
Inhibition of miR‐195 expression abolished the effects of 5‐AzaC treatment on PCa cell proliferation. PC‐3 and DU145 cells were treated with 5‐AzaC, followed by transfection with miR‐195 inhibitor or miR‐NC. (A) The expression of miR‐195 was determined using quantitative real‐time PCR. Cell proliferation was assessed using CCK‐8 assay in (B) PC‐3 and (C) DU145 cells. (D) Cell apoptosis was determined using Annexin V/PI double staining with flow cytometry. The data are presented as the mean ± SD; *n* = 3; ***P < 0.001, compared with control; ^#^ *P* < 0.05, ^##^ *P* < 0.01, compared with 5‐AzaC + miR‐NC; two‐tailed Student’s *t*‐test.

**Figure 6:**
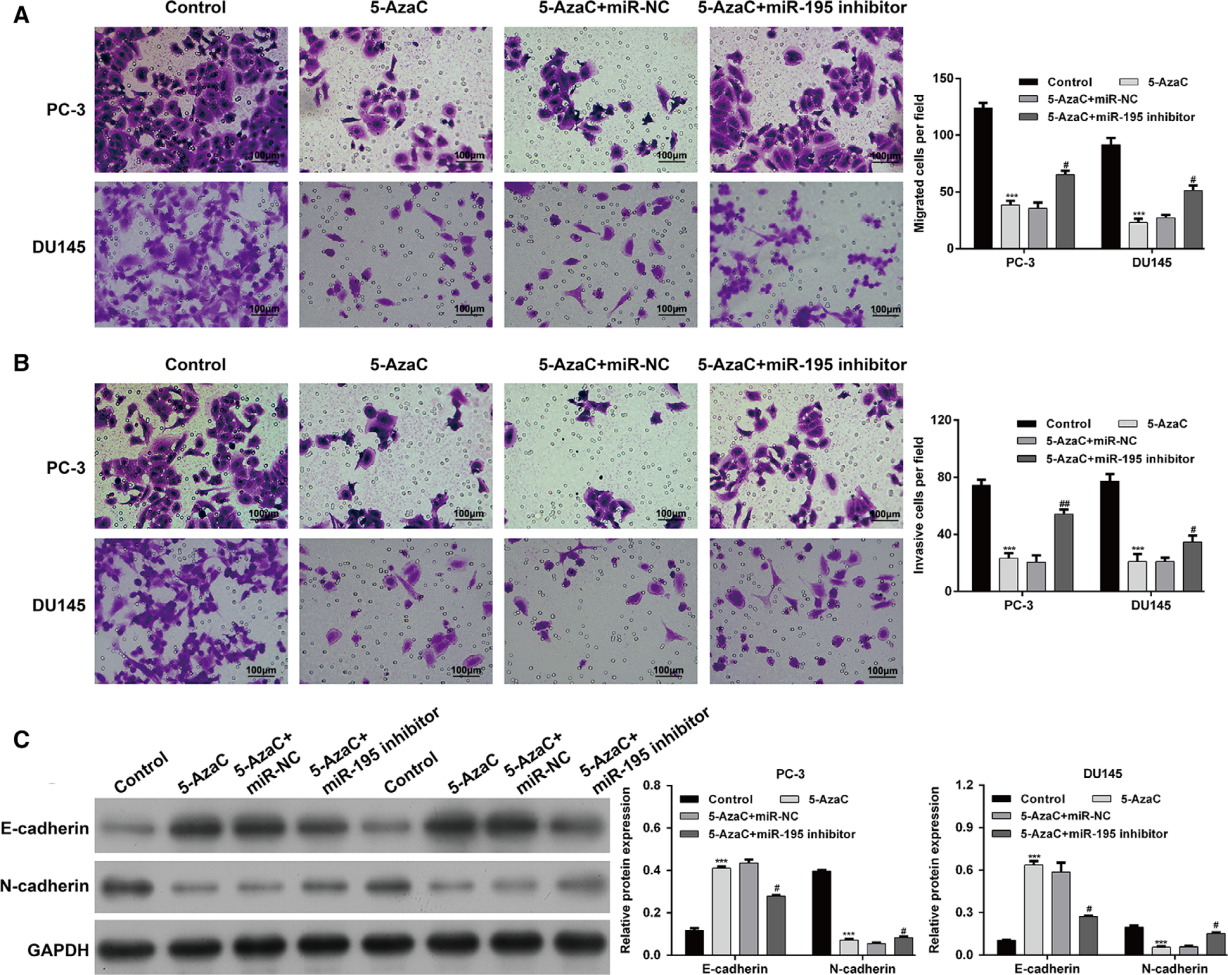
Inhibition of miR‐195 expression abolished the effects of 5‐AzaC treatment on PCa cell migration and invasion. PC‐3 and DU145 cells were treated with 5‐AzaC, followed by transfection with miR‐195 inhibitor or miR‐NC. Transwell assay was performed to evaluate (A) cell migration and (B) invasion ability in PC‐3 and DU145 cells. Original magnification ×100. (C) The protein expression of E‐cadherin and N‐cadherin was measured by western blotting. Scale bars, 100 μm. The data are presented as the mean ± SD; *n* = 3; ***P < 0.001, compared with control; ^#^ *P* < 0.05, ^##^ *P* < 0.01, compared with 5‐AzaC + miR‐NC; two‐tailed Student’s *t*‐test. GAPDH, glyceraldehyde‐3phosphate dehydrogenase.

Subsequently, we wondered whether 5‐AzaC regulated PCa cell progression through directly elevating miR‐195 expression. PC‐3 and DU145 cells were treated with 5‐AzaC, followed by transfection with miR‐195 inhibitor or miR‐NC. As shown in Fig. 5A, the expression of miR‐195 was significantly up‐regulated after 5‐AzaC treatment alone (*P* < 0.001), but notably decreased after miR‐195 inhibitor transfection in both PC‐3 and DU145 cells (*P* < 0.01). As expected, a series of functional assays, including CCK‐8 (Fig. 5B,C), flow cytometry (Fig. 5D) and transwell assays (Fig. 6A,B), demonstrated that the inhibition of cell proliferation, migration and invasion, and the increased cell apoptosis after 5‐AzaC treatment were remarkably reversed by miR‐195 inhibitor transfection in PC‐3 and DU145 cells. We also demonstrated that 5‐AzaC treatment enhanced the expression of Bax and decreased the level of Bcl2. However, the effect on the expression of Bax and Bcl2 was reversed by miR‐195 inhibitor transfection in PC‐3 and DU145 cells. Moreover, the suppressive effects of 5‐AzaC on EMT markers (E‐cadherin and N‐cadherin) were abolished by miR‐195 down‐regulation in PC‐3 and DU145 cells (Fig. 6C). These data further suggest that down‐regulated miR‐195 promoted PCa cell progression associated with DNA methylation of its promoter.

## Discussion

In this study, we observed that miR‐195 expression was dramatically decreased in PCa tissue and cell lines. In addition, enforced expression of miR‐195 potentially blocked cell proliferation, migration, invasion and EMT in PCa cell lines, PC‐3 and DU145. These results suggest that miR‐195 may serve as a tumor suppressor in PCa. Despite different PCa cell lines, our data were consistent with the studies from Cai *et al*. 27, 30, Guo *et al*. 28 and Liu *et al*. 29.

Related studies showed methylation in CpG islands could epigenetically regulate the transcription of miRNAs 34, 35. Another related study showed that down‐regulation of miR‐195 in HCC is attributed, maybe not all but at least in part, to the hypermethylation of CpG sequences in its promoter 33. Here, we further explored whether decreased miR‐195 expression was ascribed to higher DNA methylation level of promoter region by MSP analysis. As expected, hypermethylation of the promoter region of miR‐195 may be an important mechanism mediating its down‐regulation in PCa.

Unlike mutation, DNA methylation can be reversed, similar to other physiological biochemical modifications 36. 5‐Aza‐dC as an approved demethylating agent by the U.S. Food and Drug Administration underscores the usefulness of epigenetic therapy by inducing tumor cell growth and apoptosis 37, 38. Based on the study by Ghoshal *et al*. 39, intraperitoneal injection of 5‐AzaC completely attenuated growth of a transplanted tumor through demethylating and activating the antioxidant gene encoding metallothioneins. It has been shown that miR‐1 is expressed at low levels in the HCC cell line, whereas 5‐AzaC‐mediated overexpression of miR‐1 could reverse many characteristics of cancer cells, including growth, colony formation and resistance to apoptosis 36. Recently, 5‐AzaC as a demethylating agent has been used for regulating specific genes in PCa 40, 41, 42. Consistently, we further demonstrated that the suppressive impact of 5‐AzaC on the proliferation, migration and invasion ability of PCa cells may be partially because of demethylation and reactivation of miR‐195.

In summary, this study confirmed that decreased miR‐195 expression in PCa tissues and cell lines was linked to promoter methylation status. These results further emphasize that miR‐195 is modulated by epigenetic mechanisms and functions as a crucial tumor suppressor in PCa, which might offer another molecular mechanism for PCa pathogenesis.

## Conflict of interest

The authors declare no conflict of interest.

## Author contributions

XW conceived and designed the experiments. XM, LZ, ZC, XL and LW performed the experiments. XM and LZ analyzed the data. XW and XM wrote the paper. All authors read and approved the final manuscript.

## Supporting information


**Fig. S1.** (A) The expression of Bax and Bcl2 proteins in PC‐3 and DU145 cells transfected with miR‐195 mimics or control was analyzed by western blot. (B) PC‐3 and DU145 cells were treated with 5‐AzaC, followed by transfection with miR‐195 inhibitor or miR‐NC. The expression of Bax and Bcl2 proteins in PC‐3 and DU145 cells transfected with miR‐195 mimics or control was analyzed by western blot. GAPDH, glyceraldehyde‐3phosphate dehydrogenase.

**Fig. S2.** The expression of miR‐195 was determined in PC‐3 and DU145 cells treated with 5‐AzaC using quantitative real‐time PCR analysis. The data are presented as the mean ± SD; *n* = 3; ***P < 0.001, compared with control; two‐tailed Student’s *t*‐test.
