# Supplementary figures and images for: Demethylation of miR‐195 suppresses prostate cancer cell proliferation, migration and invasion
Source: FEBS Open Bio. 2020 Mar 9;10(4):525–34. doi: 10.1002/2211-5463.12799 (PMC7137791; doi:10.1002/2211-5463.12799)

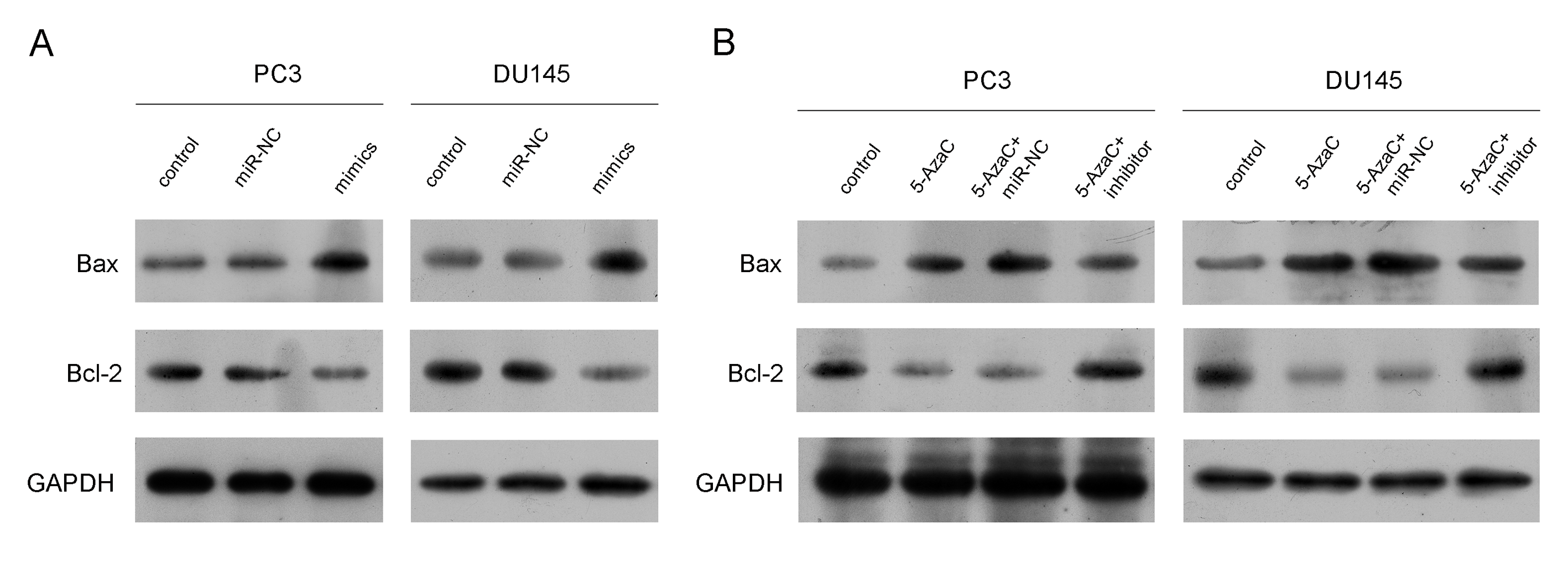

Supplement: Supplementary file 1 — Fig. S1. (A) The expression of Bax and Bcl2 proteins in PC‐3 and DU145 cells transfected with miR‐195 mimics or control was analyzed by western blot. (B) PC‐3 and DU145 cells were treated with 5‐AzaC, followed by transfection with miR‐195 inhibitor or miR‐NC. The expression of Bax and Bcl2 proteins in PC‐3 and DU145 cells transfected with miR‐195 mimics or control was analyzed by western blot. GAPDH, glyceraldehyde‐3phosphate dehydrogenase. [file FEB4-10-525-s001.tif]

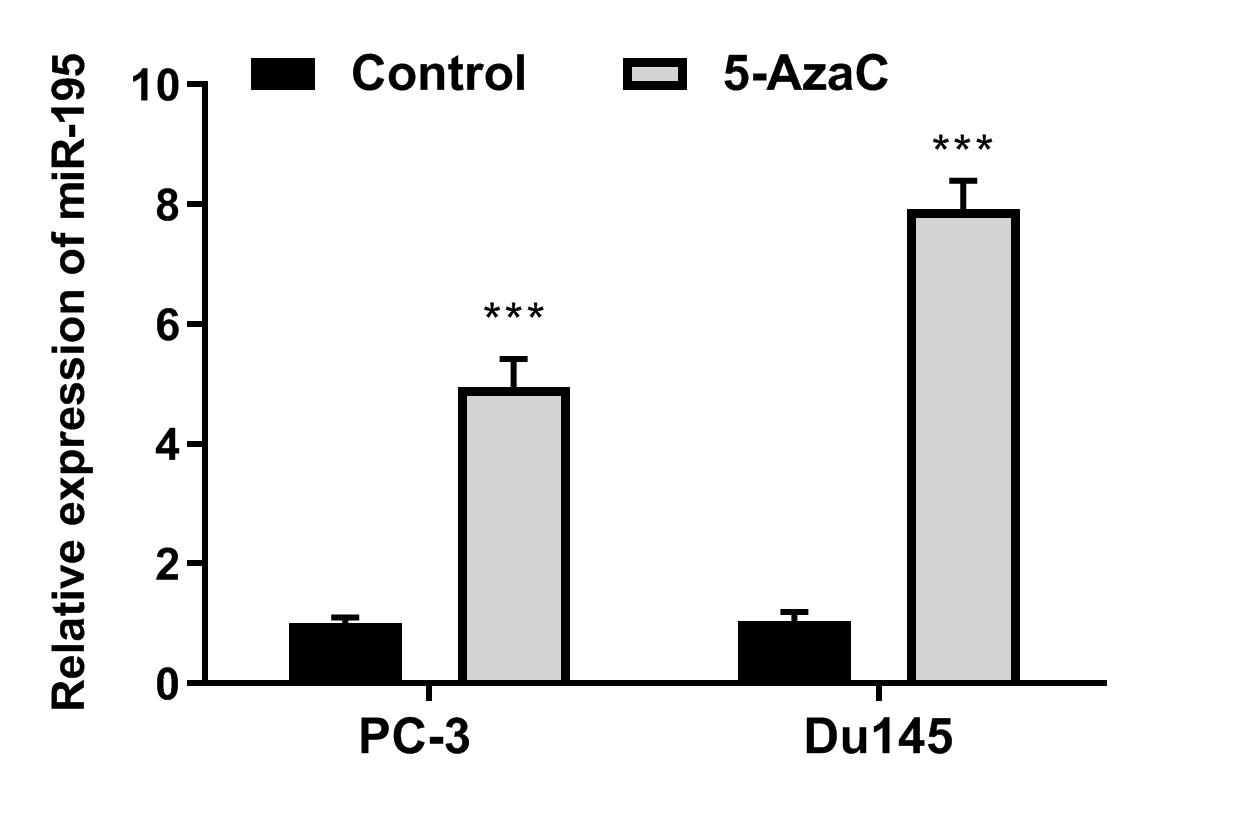

Supplement: Supplementary file 2 — Fig. S2. The expression of miR‐195 was determined in PC‐3 and DU145 cells treated with 5‐AzaC using quantitative real‐time PCR analysis. The data are presented as the mean ± SD; n = 3; ***P < 0.001, compared with control; two‐tailed Student’s t‐test. [file FEB4-10-525-s002.jpg]
